# Editorial: Cell-Matrix Mechanobiology in Diseases and Development

**DOI:** 10.3389/fmolb.2022.872969

**Published:** 2022-03-16

**Authors:** Bhumika Shokeen, Prabhat K. Purbey, Vijaykumar S. Meli

**Affiliations:** ^1^ School of Dentistry, University of California Los Angeles (UCLA), Los Angeles, United States; ^2^ Department of Microbiology, Immunology and Molecular Genetics, University of California Los Angeles (UCLA), Los Angeles, United States; ^3^ Department of Biomedical Engineering, University of California Irvine (UCI), Irvine, United States

**Keywords:** ECM, mechanotransduction, cell-matrix, disease and development, mechanobiology

Cells respond to various mechanical stimuli in the human body, including shear stress, strain, stiffness, and pressure. In mechanotransduction, cells integrate these mechanical stimuli and convert them into biochemical signals (Vogel, 2018). Several mechanosensitive molecules and cellular components participate and relay these biomechanical signals inside the cell to influence their behavior (outside-in signaling) (Mohammed et al., 2019). In turn, cells can alter the biophysical properties of the surrounding ECM (inside-out signaling) by secreting matrix remodeling enzymes. This reciprocal interaction between the cells and their immediate milieu determines development and homeostasis. However, these forces are altered in pathological conditions, leading to dysbiosis of cell-cell and cell-ECM interactions that may promote disease progression (Ingber, 2003). In this issue, several research groups have made efforts to address one of the most critical questions in the field “how these cells perceive physical forces in physiological and pathological conditions?”

One of the significant mechanical properties of ECM is substrate stiffness that can modulate cell behavior, including proliferation, angiogenesis, and inflammatory response. Cardiac fibrosis, a common cause of heart failure, results from activation of cardiac fibroblasts leading to excessive deposition of the ECM and increased matrix stiffness. In this issue, Fan and Kassiri have comprehensively reviewed cardiac fibrosis to provide a thorough discussion on the strategies to combat fibrosis and sex differences in this disease.

The importance of the surrounding ECM in determining human mesenchymal stem (hMSCs) cell fate has been relatively well studied. In their article, Meng et al. have demonstrated that mechanical properties of hMSCs and the organization of actin cytoskeleton altered by the surrounding ECM (outside-in signaling) can be used to determine their lineage commitment towards adipogenic or osteogenic fate much earlier. This incredible work showed that the mechanical properties and topography of the surrounding ECM are altered in the vicinity of the cells differentiating toward the osteogenic lineage (inside-out signaling). This study is a perfect example of reciprocal interaction between a cell and its surrounding ECM. On the other hand, due to poor cell-cell and cell-ECM interactions, stem cells have poor retention and may lose their function and ability to mediate tissue repair at the transplanted site. A comprehensive review by Shafiq et al.discusses biomaterial-based strategies that can enhance stem cell function by better retention at the transplantation site. In addition, they discuss biomaterials to deliver bioactive signals to improve stem cell function. Taken together, the discussed articles argue that ECM properties surrounding the stem cells can provide instructive cues that determine their subsequent lineage commitment, improve stem cell function, and enhance tissue regeneration.

Integrins cannot be overlooked in a discussion involving cell-ECM interaction; they help the cells interact with the ECM microenvironment. Integrins are heterodimeric transmembrane receptors that play a vital role in mechanosensing by transmitting signals inside the cell (outside-in signaling). Integrins mediate interactions between cells and cells-ECM, and their expression varies significantly between normal and tumor cells. Hou et al. has systematically discussed the role of integrins and how they influence other signaling pathways in the metastatic progression of gastrointestinal cancer.

Another mechanical cue that cells experience is geometry. Recent findings have shown that cells respond to local geometry as an extracellular cue. One of the fundamental local geometry is the surface curvature that determines the spatiotemporal organization of cells and tissues. Jin et al. built chips with cell-scale tubular (convex and concave) surfaces to address how airway smooth muscle cells (ASMCs) adapt to the cylindrical curvature. Interestingly, the ASMCs organized very differently on the concave and convex surfaces. However, upon culturing the ASMCs on tubular surfaces, they underwent phenotype transition both on concave and convex surfaces. This knowledge will help understand ASMCs pathophysiology and design artificial scaffolds.

Is the tissue surrounding the highly loaded tissues such as bones or tendons mechano-responsive? Klatte-Schulz et al. investigated the role of the subacromial bursa. The bursa is the slippery sac of fluid that facilitates the gliding motion and reduces friction between the surfaces. They found that bursa-derived cells activated mechanotransduction pathways in response to mechanical loading. This vital work can help understand the role of the bursa in the development and healing of shoulder pathologies.

Together, the insights provided by these articles would help advance the field of “Cell-Matrix Mechanobiology” and design better treatment strategies for diseases and disorders in the future.
